# Treatment priorities from the perspectives of people with dementia with Lewy bodies: a reflexive thematic analysis

**DOI:** 10.3389/frdem.2026.1721320

**Published:** 2026-04-01

**Authors:** Paula Sinead Donnelly, Kathryn Mitchell, Noleen K. McCorry, Marco Boeri, Anthony Peter Passmore, Joseph P. M. Kane

**Affiliations:** 1 Centre for Public Health, School of Medicine, Dentistry and Biomedical Sciences, Queen’s University Belfast, Belfast, United Kingdom; 2 Holywell Hospital, Antrim, United Kingdom; 3 Patient-Centered Outcomes, OPEN Health, London, United Kingdom

**Keywords:** dementia with Lewy bodies, Lewy body dementia, patient perspective, qualitative research, reflexive thematic analysis

## Abstract

**Introduction:**

Treatment priorities in dementia with Lewy bodies (DLB) have important implications for outcome selection and measurement in clinical trials and for person-centered care. The reasons underlying these priorities remain unclear. This study explored these reasons from the perspectives of people with DLB.

**Methods:**

A qualitative component was embedded within a larger stated-preference study. As part of orally administered surveys, eight people with DLB answered an open-ended question regarding which symptoms they considered most important to treat and why. Responses were analyzed using reflexive thematic analysis.

**Results:**

Six themes were identified: (1) Cognitive and functional decline as a threat to autonomy, safety, and self, (2) Motor symptoms and pain as barriers to identity, activity, and wellbeing, (3) Autonomic symptoms as sources of stigma, shame, and disruption, (4) Sleep as a foundation for functioning in daily life, (5) Neuropsychiatric and psychological symptoms and the erosion of connection, motivation, and self, and (6) Navigating symptom complexity and uncertainty.

**Discussion:**

Symptoms were prioritized for their frequency and severity, and for the ways in which they threatened valued aspects of life, such as autonomy, safety, intimacy, and sense of self. Participants’ reasoning therefore drew attention to relatively understudied dimensions of living with DLB. Although the sample was modest, participants’ accounts provided rich insights into the lived experience of DLB and its consequences for this under-researched population. The findings provide evidence to guide outcome selection in trials and highlight the need for outcome measures that capture multidimensional domains reflecting fundamental human needs, ideally through DLB-specific instruments.

## 1 Introduction

Dementia with Lewy bodies (DLB), together with Parkinson’s disease dementia (PDD), comprise Lewy body dementia (LBD), the second most common neurodegenerative dementia (Savica et al., 2013; Vann Jones and O’Brien, 2014; Hogan et al., 2016). Individuals with DLB may present with symptoms across cognitive, neuropsychiatric, motor, autonomic, sleep, psychological and behavioral domains (McKeith et al., 2017). The disorder is highly heterogeneous, with symptom constellations differing between individuals. In addition, natural fluctuations in symptom expression contribute to intra-individual variability, with symptoms varying over time within a given patient. Although DLB and PDD overlap clinically, important differences exist. DLB is clinically differentiated from PDD by the temporal sequence of symptom onset, with dementia preceding parkinsonism or occurring within 1 year of its onset in DLB, and developing more than 1 year after the onset of parkinsonism in PDD (McKeith et al., 2017). DLB may also be associated with faster motor decline than PDD (Choudhury et al., 2023) and may respond less favorably to dopaminergic treatment (Molloy et al., 2005). However, some individuals with DLB may never develop parkinsonism, whereas parkinsonism is required for a diagnosis of PDD (McKeith et al., 2017). Differences in domain-specific patterns of cognitive impairment have also been observed. For example, individuals with DLB may demonstrate greater memory impairment than those with PDD at comparable levels of global dementia severity, whereas those with PDD may demonstrate greater impairment in executive functioning (Smirnov et al., 2020). Compared with other dementia subtypes, DLB has a poorer prognosis (Mueller et al., 2017), imposes greater care partner (CP) stress (Huang et al., 2022; Yuuki et al., 2023), and incurs higher economic cost (Chen et al., 2019; Boland et al., 2024), yet remains relatively under-researched (D’Antonio et al., 2021).

Current therapies for DLB offer only modest symptomatic relief and are complicated by trade-offs, as treating one symptom can exacerbate another (Watts et al., 2023). For example, dopaminergic therapies may improve motor symptoms but increase the risk of psychosis (Goldman et al., 2008). This complexity, together with the heterogeneous nature of DLB, complicates outcome selection in clinical trials. To address these challenges, person-centered therapeutic development requires outcomes that reflect what matters most to people with DLB and their CPs. Capturing what is meaningful is likewise essential for developing clinical outcome measures (Food and Drug Administration, 2015; U.S. Department of Health and Human Services, Food and Drug Administration, 2022). The emphasis on lived experience aligns with regulatory priorities, exemplified by the United States (US) Food and Drug Administration’s (FDA) Patient-Focused Drug Development (PFDD) program (Chalasani et al., 2018). On October 15, 2024, a PFDD meeting was convened for DLB to foreground patient and CP perspectives in therapeutic development.

Recent qualitative research has advanced understanding of daily life with DLB (Larsson et al., 2019; Yumoto and Suwa, 2024); however, little is known about which symptoms should be prioritized in clinical trials and why from the perspectives of those directly affected. Of two recent reviews, only one identified a study focused specifically on treatment needs (Donnelly et al., 2025; Mammen et al., 2025). In that questionnaire study, participants selected, from a predefined list, the symptoms they would most likely prioritize for treatment (Hashimoto et al., 2022). While offering valuable insights, the fixed-choice questionnaire format does not allow researchers to explore participants’ explanations for these selections, such as the lived consequences of different symptoms. Both reviews also noted that few studies directly capture patient perspectives and that few focus specifically on DLB cohorts, potentially obscuring important DLB-specific insights (Donnelly et al., 2025; Mammen et al., 2025).

Related work in quality of life (QoL) and CP burden/stress has highlighted the substantial impact of symptoms in DLB, particularly noncognitive ones (van de Beek et al., 2019; Lee et al., 2018; Armstrong et al., 2024a,b). However, symptoms affecting QoL and CP stress do not necessarily align with the symptoms patients and CPs most wish to prioritize for treatment. For example, while visual hallucinations are associated with CP burden (Kanemoto et al., 2021), some patients report relative indifference (Larsson et al., 2019). These inconsistencies highlight the need to explore outcomes valued by patients and CPs directly, rather than inferring them from constructs such as QoL. More recently, a core outcome set (COS) for DLB trials has been developed in consultation with patients and CPs, which identified eight priority outcomes: delusions/paranoia; fluctuations in cognition, attention, and arousal; functioning; global cognition; hallucinations; QoL; motor parkinsonism; and rapid eye movement sleep behavior disorder (Kane et al., 2025). This represents important progress in aligning trial outcomes with lived experience, yet further work is needed to establish the relative importance of these outcomes and the reasons underlying their prioritization.

To address this evidence gap, we conducted a multifaceted study guided by the research question, “Which symptoms would individuals with DLB and their CPs most like to see improved by a potential therapy?” The primary component was a stated-preference web survey to elicit and quantify treatment preferences and priorities (results will be reported elsewhere). For individuals with DLB unable to complete the web survey, an orally administered survey was used, incorporating an exploratory qualitative component. This component was designed to complement and extend the quantitative findings by providing an interpretive account of how individuals with DLB discussed and framed the importance of symptoms for treatment. Here, we report the qualitative analysis, which aimed to explore participants’ accounts of why particular symptoms were prioritized for treatment. Through this exploration, the study sheds light on relatively understudied experiential dimensions of living with DLB.

## 2 Materials and methods

### 2.1 Research design

A critical realist ontological position was taken, which holds that an objective reality exists but can only ever be partially known because perceptions are mediated by language, culture, and social context (Bhaskar, 2008; Fletcher, 2017). As such, participants’ accounts are understood as situated interpretations of an underlying reality (Braun and Clarke, 2022b). A contextualist epistemology was applied, recognizing knowledge as context-dependent (Braun and Clarke, 2022b). Accordingly, accounts were viewed as contextually situated insights into both living with DLB and reasons for treatment preferences. Responses were analyzed using reflexive thematic analysis (RTA) to generate insights into the views and experiences of people with DLB, while acknowledging the researcher’s active interpretive role in data collection and analysis (Braun and Clarke, 2006, 2019, 2021a, 2022a, 2024).

### 2.2 Participants

Participants were not directly recruited into the orally administered survey but self-selected this mode by using a “help” button within the web survey. The web survey recruited a convenience sample of people with DLB and their CPs through advocacy organizations, the Join Dementia Research Network, and social media. Eligibility criteria included being aged 18 years or older, and either having a diagnosis of DLB (including prodromal DLB) according to established criteria (McKeith et al., 2017) or being a current or former informal CP of someone with DLB. Respondents also needed to be proficient in English and able to provide informed consent. Individuals in full-time residential care were excluded.

The orally administered survey was limited to participants with DLB. CPs could be present to support participation, but no data were directly collected from them. Recruitment ran from May 22, 2024, to May 7, 2025, during which eight individuals opted in. Participants were predominantly male and located in the United Kingdom (Table 1).

**Table 1:** Characteristics of participants in the orally administered survey.

Pseudonym	Gender	Age (years)	Living location	Years since diagnosis	Race
1	Female	62	USA	<1	White
2	Female	81	USA	<1	White
3	Male	55	UK	1 to <2	White
4	Male	62	UK	4 to <5	White
5	Male	75	UK	<1	White
6	Male	53	UK	8 to <9	White
7	Male	64	UK	4 to <5	White
8	Male	77	UK	<1	White

United States of America (USA); United Kingdom (UK).

Because the number of data instances (i.e., sample size) that will provide adequate data for RTA cannot be predetermined (Braun et al., 2022), no formal target was set. RTA offers flexibility in relation to dataset size, which was valuable for this study as participants were not actively recruited to the orally administered survey. The concept of saturation was not applied, as it is not compatible with RTA (Braun and Clarke, 2021b). While the notion of information power is more appropriate (Malterud et al., 2016; Braun and Clarke, 2021b), it was not used here, as participation was driven by demand for a more accessible survey mode rather than by monitoring data richness.

### 2.3 Data generation

Data were generated through an open-ended question embedded in the otherwise quantitative survey. Following a brief introduction and collection of sociodemographic and clinical characteristics, participants completed six best-best scaling choice sets (a variant of best-worst scaling) to identify the symptom domains they considered most important for treatment. They were then invited to elaborate on which symptoms mattered most and why. Although the open-ended question was the primary source of qualitative data, a conversational approach was taken throughout the survey, and relevant supplementary insights provided elsewhere were incorporated into the analysis and are reflected in the results. Participants were not given the opportunity to review their transcripts prior to analysis.

Surveys were conducted by the first author (PSD) via Microsoft Teams, audio-recorded, and automatically transcribed using its built-in feature. PSD had no prior relationships with participants and no contact beyond the survey sessions. They brought training in qualitative interviewing and analysis. Anonymized clean verbatim transcripts were prepared by PSD, with pseudonyms applied. The dataset comprised eight transcripts, with responses ranging from single paragraphs to several, and was sufficient to support interpretive analysis. Although the overall survey duration was recorded, it primarily reflected completion of the quantitative components. Given the focus here on the qualitative data, response durations are not reported.

### 2.4 Analytic approach and process

Analysis followed the six stages of RTA: (1) familiarization, (2) generating initial codes, (3) generating themes, (4) reviewing potential themes, (5) defining and naming themes, and (6) writing up (Braun and Clarke, 2006, 2022b). Analysis was primarily inductive, with themes generated from participants’ accounts, but bounded deductively by the focus of the question to ensure alignment with the study aim. Reporting adheres to the RTA Reporting Guidelines (RTARG) (Braun and Clarke, 2024). Analysis was conducted by the first author (PSD), with regular discussion and critical input from a second author (JK).

PSD began by immersing themselves in the data, reading and rereading transcripts and noting early reflections. An early observation was that symptoms were prioritized for reasons beyond their severity or frequency, often linked to their wider impact on the person. These early insights informed, but did not constrain, subsequent coding.

Coding was carried out in Microsoft Word, with each data item entered into a table containing one column for data and one for codes. Coding was recursive, with codes developed, refined, and revised. Both semantic and latent coding were applied, meaning data could be assigned multiple codes. For example, “…then what is the purpose? You might as well euthanize me. There just is no purpose” was coded semantically as “expresses desire for euthanasia” and latently as “cognitive and/or functional decline threatens sense of self-worth”. Codes were reviewed and discussed with the second author (JK) to identify any new insights.

Codes were then grouped into candidate themes in Microsoft Excel. Themes, defined in RTA as patterns of shared meaning organized by a core concept (Braun and Clarke, 2019), were generated inductively and organized around symptom domains to provide a framework for presenting participants’ accounts. Consistent with RTA, themes and subthemes were determined by their analytic relevance to the research question rather than by recurrence (Braun and Clarke, 2021b). Given the iterative nature of RTA (Braun and Clarke, 2006, 2022b), codes were revisited at this stage and, where necessary, collapsed, revised, or removed if no longer relevant to the analysis. For example, codes relating to care experiences unrelated to the research question did not progress and were removed (e.g., “tests ruled out Alzheimer’s disease”; “doctor dismissed need for assessment”). At this point, approximately 151 codes were retained. Once all codes had been grouped into candidate themes, these were discussed by PSD and JK to interrogate their meaning. Where subthemes were too thin, these were collapsed into broader themes. For example, codes related to memory and functioning were combined into a single theme. Finally, PSD revisited the data to ensure that the final themes provided a structured interpretation of participants’ accounts.

Examples of data extracts with their codes and final theme are provided in Table 2. Frequency of themes, interrater reliability, and consensus coding were not employed, as these practices are inconsistent with RTA, which emphasizes the researcher’s active and interpretive role in analysis and the generation of themes.

**Table 2:** Examples of data extracts and coding with themes.

Data extract	Coding	Themes
What symptoms are most important? I think just being able to think clearly [c1]. To be able to plan out your day, to not forget to turn off the stove (laughs) [c2, c3]. To remember to do your activities of daily living [c4]. You know, it’s just kind of basic functioning [c5, c6]. And then to regulate your sleep [c7]. Like you cannot do the other if you cannot sleep at night, or if you cannot fall asleep, or if you cannot wake up [c8, c9].	c1. Thinking clearly is important c2. Want to be able to plan the day c3. Fear of memory-related safety risks c4. Important to remember to do ADLs c5. Basic functioning is important c6. Desire to manage daily activities independently c7. Regulating sleep is important c8. Sleep is foundational for daily functioning. c9. Inability to sleep disrupts ability to function	C1-6: Cognitive and functional decline as a threat to autonomy, safety, and self C7-9: Sleep as a foundation for functioning in daily life
The autonomic dysfunction. The first time incontinence happened I was devastated [c38]. I thought I would not ever be able to go away from home, that I’d be homebound the whole time [c39], and so when we solved some of the problems, that was good [c40]. But I worry about them coming back [c41] because it’s a surprise every day you know [c42]. The phrase that we use here, I do not know if you use it, it’s called a “Lewy body dementia roller coaster” [c43]. And it’s because out of nowhere you are having a good day and all of a sudden things happen and you did not see it coming. I get up. It’s getting where I just wake up and I’m going to have a bad day, and I do not know why [c44].	c38. Devastation experiencing incontinence c39. Fear of being housebound with incontinence c40. Happy when incontinence was resolved c41. Worry about incontinence returning c42. Daily uncertainty related to symptoms c43. Lewy body dementia roller coaster c44. Unpredictable day-to-day experience	C38-41: Autonomic symptoms as sources of stigma, shame, and disruption C42-44: Navigating symptom complexity and uncertainty

### 2.5 Reflexive/positionality statement

RTA recognizes and values researcher subjectivity as integral to knowledge production (Braun et al., 2022), making reflexivity a core element of the analytic process. Reflexivity involves ongoing internal dialogue and reflection on one’s positionality as a researcher (Berger, 2015; Braun et al., 2022). Here, we provide a brief positionality statement, with a fuller reflexive account from the first author in the Supplementary material. The research team brought together experience of knowing individuals affected by DLB, alongside clinical and research expertise in geriatric psychiatry, Lewy body dementia, and health psychology.

### 2.6 Ethics

The study was approved by the Faculty of Medicine, Health and Life Sciences Research Ethics Committee at Queen’s University Belfast in March 2024 (MHLS 23_164).

Participants entered the study via the web survey, where consent was formally obtained through an embedded form. Participants who opted for the orally administered survey did so via the web survey and therefore provided written consent prior to participation. Consent was verbally reconfirmed before commencement of the oral survey. This followed a review of the participant information sheet, which was read aloud and displayed to remind participants of the study aims, nature of participation, potential benefits and risks, and their right to withdraw at any time. Participants were informed that data could be withdrawn for up to 1 week after participation, after which all data were anonymized.

A protocol aligned with the legal requirements of the Mental Capacity Act (MCA) 2016 was prepared in case it became apparent that a participant lacked capacity to give informed consent. All participants completed the oral survey without invoking this protocol.

## 3 Results

**Figure 1:**
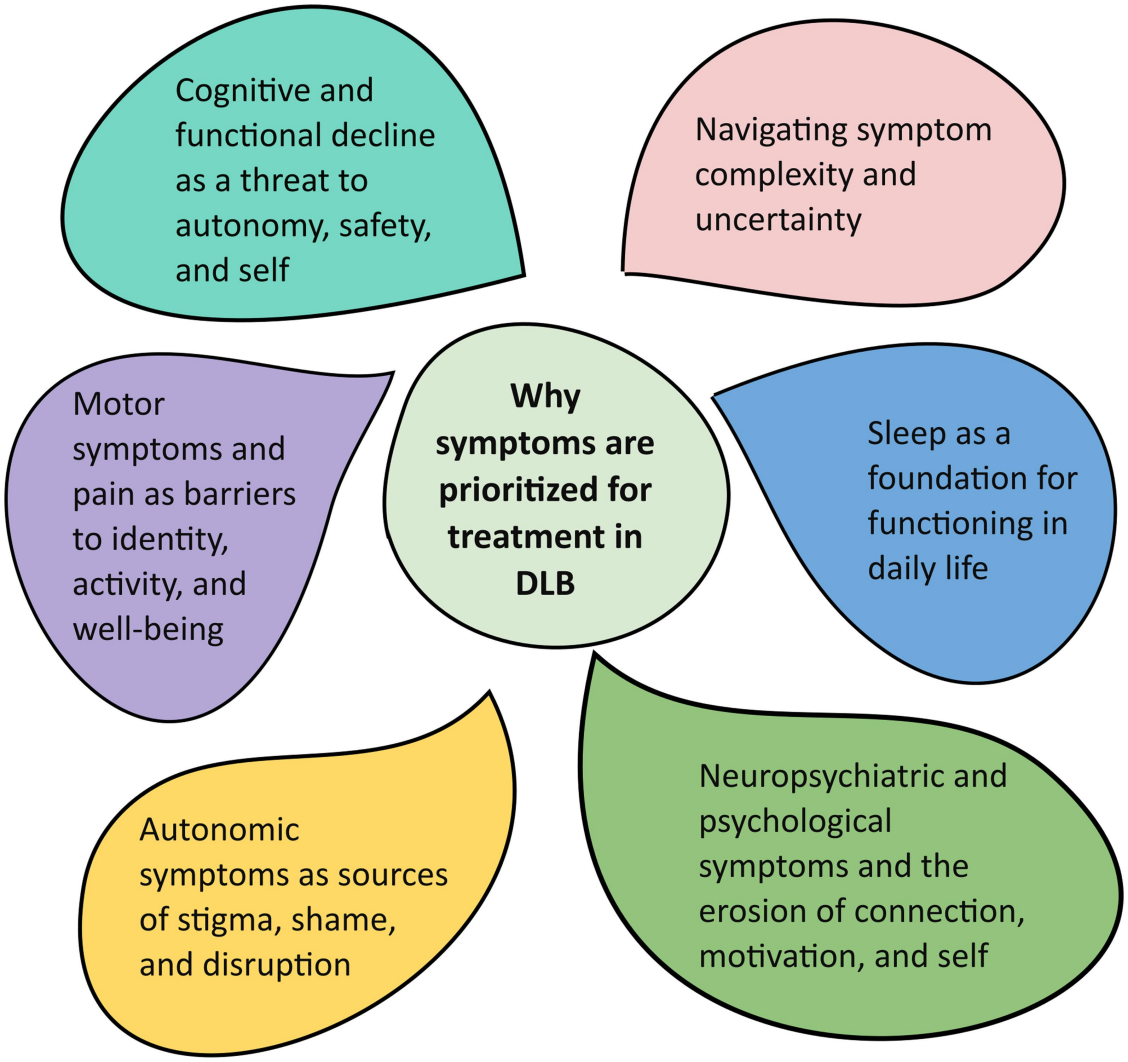
Themes illustrating the reasons for symptom treatment priorities among people with DLB.

Six themes were generated from the data (Figure 1), each illustrated with anonymized quotations from participants. No rank order is implied among the themes. Although presented separately, the themes are interrelated, with some ideas appearing across domains. Each theme, however, captures a distinct pattern of meaning organized around a central concept, contributing to a broader account of how participants prioritized symptoms for treatment.

### 3.1 Cognitive and functional decline as a threat to autonomy, safety, and self

This theme examines how participants accounted for cognitive and functional symptoms as priorities for treatment. In some accounts, these symptoms disrupted not only daily routines but also autonomy, safety, self, and purpose. For example, one participant described how cognitive clarity shaped their capacity to manage everyday life:

*“…just being able to think clearly. To be able to plan out your day. To not forget to turn off the stove.”* (P1)

For this individual, cognitive clarity was essential for both managing practical tasks and sustaining a sense of safety and stability. When clarity was compromised, everyday tasks could feel precarious. Another participant described memory difficulties as disruptive and emotionally taxing due to their constant recurrence:

*“…it is the thing that troubles me the most… on a daily basis, or an hourly basis…that for me is the most, and I'm going to use the word ‘traumatic’, because I think it is traumatic for me.”* (P4)

The use of the term “traumatic” highlighted the psychological weight of these experiences, showing their impact on emotional wellbeing beyond mere recurrence. For Participant 7, longstanding cognitive difficulties present since birth were compounded by dementia, reinforcing the prioritization of cognitive symptoms for treatment. Elsewhere, cognitive decline was described as threatening meaning and value in life:

*“If I am not serving any purpose in life, if I'm not accomplishing anything or able to carry out conversations…, then what is the purpose? You might as well euthanize me. There just is no purpose.”* (P1)

Such accounts illustrate how loss of cognitive and functional abilities can evoke existential concerns. They resonate with literature describing dementia as an existential threat, where compromised identity, independence, and social participation undermine someone’s sense of meaning and purpose (Cheston et al., 2015). As personhood in dementia is often affirmed through interpersonal connection (Hennelly et al., 2021), diminished capacity to communicate or contribute can erode a sense of value and self. Prior work in DLB similarly highlights how selfhood is tied to providing for others and avoiding the sense of being a burden (Larsson et al., 2019).

In some cases, it was not only the current symptoms but also perceptions of their inevitable progression that intensified concerns. For participant 7, reminders of inevitable decline were triggered unexpectedly, for instance when hearing about others with dementia who had died:

*“….sometimes I’m going along thinking ‘I’m just normal. I’ve not got dementia,’ but when I hear things come out from the blue that it's a wee little, you know, a reminder I’ve got it. That worries me.”* (P7)

Such “reality checks” disrupted psychological equilibrium, confronting a temporary sense of normality and intensifying anticipatory anxiety, echoing findings from other studies of DLB/LBD (Bentley et al., 2021; Yumoto and Suwa, 2024). Similarly, participant 2 reflected on the desire to preserve cognition for as long as possible, while deliberately avoiding thoughts of inevitable decline:

*“I want to stay as aware as I can and I know it can't be forever, so I try not to think about the things that I think I have no control. I try to not just think about them all the time, but you can’t do that forever.”* (P2)

For these participants, both the experience and anticipation of decline associated with the dementia syndrome were destabilizing. Avoidance-oriented coping strategies, commonly observed among people with dementia (Bjørkløf et al., 2019), provided a temporary means of managing this uncertainty.

Taken together, these accounts illustrate that cognitive and functional symptoms were prioritized not only for their immediate impact on daily life, but also for how they shaped participants’ sense of identity, anticipated future, and purpose.

### 3.2 Motor symptoms and pain as barriers to identity, activity, and wellbeing

This theme explores why motor symptoms were prioritized for treatment. While experienced as physical impairments, these symptoms also disrupted valued routines, identity, and emotional wellbeing. Their salience often arose when they interfered with previously valued or habitual activities, reshaping participants’ sense of self.

One participant described the personal impact of reduced mobility:

*“I’m piling on weight because I can’t exercise. I’ve still got quite a lot of my faculties left, so it’s my mobility [that] is restricting me enjoying my life. So mobility is important for me.”* (P6)

For this participant, motor symptoms were prioritized because they had become the main barrier to enjoying life. This aligns with evidence that physical activity promotes emotional wellbeing and QoL in dementia (Farina et al., 2021; Telenius et al., 2022). The mention of weight gain also suggested a compounding effect, in which reduced mobility curtailed activity, leading to weight gain that further limited mobility.

For others, the meaning of motor symptoms lay less in physical health than in the disruption of valued roles and activities. Recollections of past abilities highlighted both physical loss and its emotional consequences, with participants’ accounts reflecting a loss of freedom and independence:

*“Problems with my sense of gravity and constantly worrying about falling. It affects my speech so that I developed a stutter. Its stopped me from physical exercise. I used to do table tennis, and I used to run marathons. I can’t risk getting lost or falling. That was… that’s been tough.”* (P8)

*“I put a fence up right round the house two or three years ago…It was a huge fence. I did that myself. I put every screw in the thing. I dug every hole for the posts and everything. I just couldn't even think of doing something like that now… I'd like to say that the spirit was willing, but the flesh is weak. But you know that's not the case. They're both weak.”* (P5)

Such accounts resonate with previous findings that the most troubling symptoms in DLB were those that limited activity and social participation (Larsson et al., 2019). In the present study, participants drew on memories of past ability to emphasize the depth of present loss. For both participants, physical activity was central to identity, not just a pastime. In participant 8’s case, precautionary withdrawal from activities due to fear of falling reflected findings that fear often led people to remain at home, further reducing QoL (Larsson et al., 2019).

Beyond physical loss, some participants described distress linked to the social visibility of motor decline:

*“when you go out or you… you’re obviously ill. The shuffle walk that you develop is embarrassing.”* (P8)

Here, the change in gait was distressing not only for its physical effects but also for what it signaled socially. The participant’s sense of shame reflects the internalized and private consequences associated with the stigma surrounding dementia (Aldridge et al., 2019; Lopez et al., 2020), and echoes findings that people with dementia feel scrutinized by the gaze of others (van Wijngaarden et al., 2019). Similarly, in prior DLB work, the sense of self was affected by the perceived judgment of others (Larsson et al., 2019).

By contrast, others deprioritized motor issues when they were perceived as less severe than other symptoms:

*“the movement part of it doesn't bother me as much. The tremors are just tremors, you know.”* (P1)

For this participant, the associated pain was more pressing, creating a cycle of disrupted sleep, daytime fatigue, and further sleep disturbance:

*“It’s the pain that comes with it… you start getting into this cycle of you have pain, you can't sleep, you're tired during the day, you know, so you nap and then you can't sleep.”* (P1)

This is not surprising as pain intensity has been negatively associated with QoL in dementia (Wagatsuma et al., 2021). Pain management was complicated by treatment trade-offs. Some analgesic medications were seen as impairing cognition and causing irritability, leading this participant to tolerate pain, though not without emotional cost:

*“If I take it throughout the day, I'm not going to get anything else accomplished throughout that day…*So *sometimes I will keep the pain to be able to function… that does make you maybe a little more snappy, a little less agreeable because you're in pain.”* (P1)

For this individual, symptom management was not solely about physical comfort, but about maintaining a sense of usefulness and continuity of identity through participation in functional activities. They reported preferring medical marijuana, which they felt alleviated pain without sedative effects, though prescribing practices were a source of frustration:

*“The best thing I have found for pain…is medical marijuana. It's the one thing they don't readily necessarily utilize… but they'll give you a strong muscle relaxer. That makes no sense.”* (P1)

Overall, participants’ reflections show that motor symptoms carried meaning beyond mobility itself, constraining valued roles, social participation, and QoL.

### 3.3 Autonomic symptoms as sources of stigma, shame, and disruption

This theme captures how autonomic symptoms, particularly incontinence and constipation, were prioritized for treatment through participants’ accounts of stigma, shame, and disruption to daily life. Participants described them as *“distressing,” “really embarrassing,”* and *“discomforting,”* emphasizing the emotional and social burden beyond physical discomfort. Fear of lost dignity and social embarrassment was a recurrent concern shaping why these symptoms were considered treatment priorities.

For one participant, incontinence represented a threat to freedom and social participation:

*“The first time incontinence happened I was devastated. I thought I wouldn't ever be able to go away from home, that I'd be homebound the whole time.”* (P2)

Here, incontinence was framed as both a social disruption and a marker of stigma, echoing research that describes incontinence as humiliating and embarrassing, with the potential to lead to social isolation (Cole and Drennan, 2019; Murphy et al., 2021). With incontinence recognized as a stigmatized condition (Cole and Drennan, 2019; Chatterton, 2024), participant 2’s reference to social withdrawal illustrates a process of self-stigmatization (Milne, 2010). Although this participant eventually experienced symptom relief, the unpredictability of DLB meant that the possibility of recurrence continued to provoke anxiety.

Other participants emphasized the relentless inconvenience of autonomic dysfunction. Frustration with frequent and urgent urination, disruption to sleep, and the ways in which necessary activities, such as drinking water, could exacerbate symptoms were described. Such accounts point to constant vigilance and ongoing disruption, compounding the emotional burden of these symptoms. These findings align with evidence that patient-reported QoL in DLB is correlated with autonomic symptom burden (Armstrong et al., 2024b).

By contrast, some autonomic symptoms, such as cold extremities, were described as manageable with practical adjustments:

*“I try to help myself if possible, and my wife helps me as much as she can, and we can deal with some of these things ourselves to a certain extent. I don't know how I will deal with them when it gets worse but. As long as we can deal with them sort of. They sort of take a back seat if you see what I mean.”* (P5)

This suggests that perceived controllability influenced prioritization. Support from a CP and adjustments like warmer clothing enabled symptoms to be reframed as tolerable. In terms of Lazarus and Folkman’s Transactional Model of Stress and Coping (Lazarus and Folkman, 1984), the participant’s primary appraisal appeared to classify these symptoms as potentially threatening, while secondary appraisal involved evaluating the practical and relational resources available to manage them. The ability to draw on available resources led to the use of problem-focused coping (e.g., practical adjustments or CP support). Together, these strategies reduced perceived threat and lowered treatment priority.

Even so, the participant recognized that coping resources might be insufficient as the condition progressed. The anticipation of decline itself was distressing, with the participant highlighting the fear of reaching *“that stage”* of bedwetting. This demonstrates how even the prospect of worsening autonomic symptoms evoked anxiety and influenced treatment priorities.

### 3.4 Sleep as a foundation for functioning in daily life

The analysis identified sleep as foundational to daily functioning, as described by Participant 1:

*“to regulate your sleep. Like you can't do the other [basic functioning] if you can't sleep at night, or if you can't fall asleep, or if you can't wake up.”* (P1)

This resonates with prior work showing that disrupted sleep undermines activities of daily living (Idalino et al., 2024), with impairments in these activities associated with poorer QoL in DLB (Toya et al., 2024). Our findings extend this by showing how participants positioned sleep as a prerequisite for functioning and a foundational resource on which other aspects of daily life were seen to depend.

Similar concerns were raised by participant 3, who described rapid eye movement sleep behavior disorder (RBD) as *“debilitating.”* However, for another, RBD behaviors had become less distressing due to habituation, though sleep remained a priority because it enabled rest and daytime functionality (P6). This account illustrates adaptation, as the participant adjusted their expectations around sleep to cope with ongoing disturbances. Such adaptation is consistent with coping strategies reported in dementia (Bjørkløf et al., 2019). The participant also emphasized feeling unrested, indicating that even when RBD behaviors themselves were no longer distressing, sleep was valued for its cumulative effects on energy and wider impact on coping in everyday life. Participants’ accounts therefore aligned with evidence of a direct link between sleep, health, and cognition (Shokri-Kojori et al., 2018; Grandner, 2020), and with research emphasizing the importance of sleep for QoL in dementia (Petrovsky et al., 2018).

### 3.5 Neuropsychiatric and psychological symptoms and the erosion of connection, motivation, and self

This theme captures how neuropsychiatric and psychological symptoms became treatment priorities when they impacted relationships, provoked distress, or altered behavior in ways that felt unfamiliar. Three subthemes illustrate these processes:

#### 3.5.1 The emotional and relational weight of hallucinations

Hallucinations were prioritized when they provoked fear or disrupted relationships, but deprioritized when perceived as benign. For one participant, visual hallucinations were described in strikingly emotive terms:

*“They were really, really, really scary. They were the scariest thing that I can imagine…”* (P2)

This mental anguish illustrates why visual hallucinations became a treatment priority, resonating with previous findings of mental distress among people with DLB experiencing them (Yumoto and Suwa, 2021). For another participant, auditory hallucinations disrupted interpersonal trust through misattributions, creating tension with neighbors:

*“I get a lot of auditory hallucinations which drive me quite demented…especially at night. To me, that's one that I'd really like to see something done for because it affects my relationship with my neighbors and everything because I think it's my neighbors.”* (P3)

This suggests that as insight into the non-real nature of the hallucinatory content declined, the hallucinations became more concerning. By contrast, consistent with findings that some people perceive visual hallucinations as less concerning than the loss of independence or cognitive faculties (Renouf et al., 2018), participant 4 explained:

*“I am so fortunate that they're not frightening and therefore they are way down on my list, way down.”* (P4)

This may reflect greater insight on the part of the participant into the reality of the hallucinations as a symptom rather than something real, since insight is reported to influence perceived threat and associated distress (Renouf et al., 2018).

Together, these accounts show that the emotional and relational impact of hallucinations, rather than presence alone, determined whether they were prioritized.

#### 3.5.2 The loss of empathy and emotional connection

A perceived loss of empathy was described as highly concerning for participant 6, as it disrupted intimate relationships and their ability to connect emotionally with others. Despite retaining awareness of expected emotional responses, they felt unable to act on them:

*“I can't show love or support to my wife. Even though I love her, I can't do the emotional, physical thing of cuddling now or reassuring her.”* (P6)

This juxtaposition between intact emotional knowledge and the inability to act on it created a painful dissonance that unsettled the participant’s sense of self as a caring partner. The same detachment was described in the context of bereavement:

*“We lost our youngest daughter three years now. I grieved for 20 minutes and that’s it. My wife still misses her to this day. And I can see her sitting there upset sometimes. Mentally part of my brain is saying I know what I should do, but the other part of my brain is saying you can’t do that. You can’t.”* (P6)

This reflection highlights empathy as a resource for maintaining relationships (Betzler, 2019). Awareness of their spouse’s need for emotional connection compounded the participant’s distress, making the loss of empathy especially painful. Similar changes in spousal intimacy have been reported in related disorders (Vatter et al., 2018; Sandberg, 2023), though systematic exploration in DLB remains limited. Difficulties with emotional connection also extended beyond the spousal relationship, as the participant expressed discomfort with physical closeness, contrasting with their previously affectionate disposition:

*“People do it as a joke. They come up and cuddle me. I'm not—I used to be a touchy feely person. I can't have it now.” (P6)*

These changes were described as the *“biggest challenge,”* (P6) distressing not only because of their personal impact but also for their consequences for loved ones.

#### 3.5.3 Shifting motivation and self-regulation

Motivational anhedonia and changes in behavioral regulation were described as a source of frustration, particularly when they disrupt valued routines. Participant 7 noted a marked decline in their ability to sustain healthy behaviors, which contrasted with their previously active lifestyle:

*“I've put a lot of weight on. I've never been this in all my life… I feel that I want to go and do something, the thought is up there, but within a couple of seconds it just, you know, I lose that motive to go and do it…I miss all that. So the things I used to do in the past… I wish I carried on with it. But I'll never be able to.”* (P7)

The participant’s emotional response to this behavioral change can be understood through self-discrepancy theory (Higgins, 1987), which postulates that emotional responses arise when one’s actual self diverges from the ideal self (aspirations) or ought self (obligations). Here, the participant’s awareness of a growing gap between past/ideal capacities and present limitations generated dejection-related emotions. This emotional weight helps explain why these psychological changes were described as particularly challenging.

### 3.6 Navigating symptom complexity and uncertainty

This theme captures how participants experienced DLB symptoms as both interrelated and unpredictable, complicating the process of identifying treatment priorities. Several participants described how changes in one symptom could exacerbate another:

*“I think they're all so interrelated. When one messes up, the other messes up.”* (P1)

*“They’re all important. They all have a part to play in this.”* (P5)

Unpredictability was also central to daily experiences, with variability from day to day making the disease trajectory difficult to anticipate:

*“…it's a surprise every day you know…out of nowhere you're having a good day and all of a sudden things happen and you didn't see it coming…I just wake up and I'm going to have a bad day, and I don't know why.”* (P2)

For this individual, unpredictability was captured through the metaphor of the “Lewy body roller coaster,” which offered a way of reframing uncertainty as something nameable, helping them make sense of an otherwise disorientating experience.

Anticipated decline further shaped how participants thought about symptoms and their treatment. For participant 5, symptoms were not currently a major concern because they could be self-managed, but there was an awareness that progression might eventually overwhelm coping strategies:

*“I don't know how I will deal with them when it gets worse, but. As long as we can deal with them sort of.”* (P5)

As a whole, these accounts illustrate how symptom interrelatedness, unpredictability, and anticipated progression combined to create uncertainty, making prioritization complex. The findings resonate with evidence that people affected by DLB identify knowing what to expect and developing a staging system as key research priorities (Armstrong et al., 2020; Holden et al., 2023; Kinchin et al., 2025b).

## 4 Discussion

This qualitative analysis explored why people with DLB prioritized symptoms for treatment. To our knowledge, this is the first study to qualitatively explore these priorities from the perspective of people with DLB. Participants’ narratives revealed the complexity of symptom management and the emotional, relational, and personal implications of symptoms. Symptoms were not always inherently bothersome. Rather, they were prioritized for their frequency or perceived severity, or because they threatened valued aspects of life such as autonomy, safety, intimacy, and sense of self. This occurred through both their immediate impact and anticipated progression. These valued aspects reflect fundamental human needs and resonate with Maslow’s hierarchy of needs (Maslow, 1943), including threats to safety (e.g., fears of accidents or falling), disruptions to love and belonging (e.g., loss of emotional connection), and erosion of self-esteem and freedom. Symptom priorities therefore reflected not only clinical concerns but also the preservation of these needs.

Prior work in DLB found that patients most frequently selected memory impairment as the symptom they would most likely prioritize for treatment, followed by bradykinesia, abnormal posture, and constipation, whereas CPs most frequently selected memory impairment, followed by visual hallucinations and bradykinesia (Hashimoto et al., 2022). Rather than establishing a relative ranking of symptoms for treatment, this qualitative analysis complements existing work by providing interpretive insight into the processes and meanings that may underlie symptom prioritization. In that study, some differences in the symptoms selected between patients and CPs were observed (Hashimoto et al., 2022). One potential contributor is that patients experience symptoms as direct and embodied phenomena, whereas CPs encounter symptoms primarily through their behavioral, relational, and practical consequences. This difference in experiential position may influence how symptoms are experienced, evaluated, and prioritized. For instance, visual hallucinations may not distress patients (Abbate et al., 2014) yet lead CPs to feel unable to leave the person alone (Renouf et al., 2018). The degree to which an individual retains insight into their symptoms may also shape their experience and treatment priorities. Prior work has shown that greater insight into hallucinations may reduce perceived threat or distress, whereas declining insight may increase fear and distress (Renouf et al., 2018; Montagnese et al., 2022). As the current analysis focuses specifically on the patient perspective, future work is needed to explore the experiences shaping the treatment priorities of CPs. Such evidence could support clinical discussions of treatment goals, particularly where therapeutic trade-offs may be experienced differently by patients and CPs.

Analysis of participants’ reasons for prioritizing particular symptoms for treatment resonates with topics previously identified as research priorities in DLB. These topics include the development of pharmacological and non-pharmacological interventions, support and information resources, and better understanding of disease progression, the lived experience, and the impact of DLB on QoL (Armstrong et al., 2020; Holden et al., 2023; Kinchin et al., 2025b). Considered alongside these priorities, the current work contributes to understanding the lived experience of people with DLB by offering insight into how treatment priorities are shaped through the everyday experience of symptoms and their consequences. From a therapeutic development perspective, the insights suggest that therapeutic benefit may also be understood in relation to impacts on valued aspects of life, rather than symptom change alone. Similarly, in relation to research on disease progression, participants’ accounts highlighted the destabilizing effects of unpredictability. This suggests that further work on this topic could carry implications beyond classification or staging alone, as better understanding could potentially reduce some of the anticipatory anxiety described in daily life.

While the survey from which participants for this study were recruited identified which symptoms were prioritized overall (Donnelly et al., 2024), this qualitative analysis explained why they mattered by uncovering the meanings and consequences attached to them. Based on these insights, outcome assessment in trials should capture both clinical indices of symptom burden and patient-reported evaluations of how symptoms affect fundamental needs such as relationships, independence, and self-esteem. Although multidimensional measures that extend beyond clinical symptoms are often included in DLB research (Donnelly et al., 2025), few are disease-specific or validated. Prior work has shown that existing preference-based outcome measures only partially capture what people with dementia consider central to QoL, with many domains echoing participants’ priorities in this study (Engel et al., 2020; Kinchin et al., 2025a). These shortcomings are not only methodological but also ethical and economic. The limitations of existing measures mean that interventions whose benefits lie in domains such as stigma or relationships may not be advanced or may be undervalued in cost-effectiveness analyses, since these outcomes are inconsistently captured (Engel et al., 2020; Kinchin et al., 2025a). This highlights the need for outcome assessments and measurement frameworks that are sensitive to the lived experience and priorities of people with DLB.

The finding that symptoms were often prioritized not only for their frequency and severity, but for the ways in which they threatened valued aspects of life, has important clinical implications. This highlights the potential role of non-pharmacological interventions to preserve or restore valued aspects of life, even where direct symptom improvement may be modest. For example, evidence from the experience of those with progressive memory disorders suggests that participation in non-pharmacological interventions can strengthen a sense of personhood by increasing understanding of themselves with the disease, establishing dignity, and providing opportunities for challenge (Tuomikoski et al., 2022). In LBD, physical therapy, exercise programs, and occupational therapy may improve gait speed, balance, and functional ability, potentially reducing fear of falling and helping to mitigate social withdrawal (Connors et al., 2018). Cognitive rehabilitation has also been associated with improvements in depression, social involvement, and perceived self-efficacy in individuals with LBD (Hindle et al., 2018). Such approaches may additionally support the development of coping strategies by helping individuals feel better able to manage their symptoms. Participants in this study appraised symptoms differently depending on their perceived coping capacity, such as sometimes perceiving autonomic symptoms as tolerable when they could be self-managed. Consistent with the transactional model of stress and coping (Lazarus and Folkman, 1984), interventions could therefore support individuals in strengthening coping resources and identifying effective coping strategies. This further reinforces the importance of person-centered care, highlighting the need to recognize individual differences in symptom appraisal and to support coping strategies alongside therapeutic intervention.

A limitation of this study is that reliance on a single survey question may have led participants to provide relatively brief responses. Several characteristics of the study sample also shaped the scope of analysis. The self-selected sample, largely in the early–mid stages of DLB, foregrounded perspectives specific to this disease stage. Given the heterogeneous clinical presentation of DLB, treatment priorities may be diverse, and the findings presented reflect the specific symptom experiences represented within this sample. The sample was also predominantly male, and in light of evidence that symptom experiences in DLB may differ by gender (Chiu et al., 2018; Utsumi et al., 2020), the analysis may be influenced by this gender composition. The sample was racially homogeneous, and most participants were from the United Kingdom. Priorities and interpretations of symptoms may differ in more racially, ethnically, or geographically diverse groups. We did not collect data on educational attainment or Clinical Dementia Rating (CDR) staging, which limits comparison with other cohorts and constrains interpretation of findings in relation to dementia severity. Although participants reported time since diagnosis and geographic location, we did not explore thematic variation by duration or region, as the analysis focused on patterns across the dataset. Individuals in full-time residential care were not eligible to participate. As a result, the analysis reflects the perspectives of people living in the community, and the experiences, priorities, and impact of symptoms may differ for those in residential settings.

However, the richness and candor of participants’ accounts balanced the modest sample size, and active interpretive engagement supported a nuanced understanding of how treatment priorities were constructed in DLB. Recruiting people with DLB is challenging (Goldman et al., 2020; Donnelly et al., 2025), particularly for those without access to the internet. The perspectives of people with DLB are also underrepresented in research relative to their CPs (Donnelly et al., 2025; Mammen et al., 2025), highlighting the importance of capturing their voice. Moreover, by capturing the implications of symptoms for valued aspects of life, including stigma, autonomy, and intimacy, the study provides insights into relatively understudied aspects of living with DLB.

Overall, this study shows that symptoms were often prioritized not simply for their frequency or severity, but because they undermined valued aspects of life that reflect fundamental human needs. These insights suggest that evaluating therapeutic development in DLB should go beyond measuring symptom reduction alone to include multidimensional outcomes such as relationships, self-esteem, and self-concept. A more person-centered approach to outcome selection and measurement would ensure that future trials address what matters most to those affected.

## Data Availability

The raw data supporting the conclusions of this article will be made available by the authors, without undue reservation.
