# Supplementary material for: Treatment priorities from the perspectives of people with dementia with Lewy bodies: a reflexive thematic analysis
Source: Front Dement. 2026 Apr 1;5:1721320. doi: 10.3389/frdem.2026.1721320 (PMC13079032; doi:10.3389/frdem.2026.1721320)
Supplement: Supplementary file 1 [file Supplementary_File_1.docx]

Supplementary Material

# Reflexivity statement

This study was designed to explore treatment preferences and priorities in DLB, with the qualitative component intended to illuminate the reasons underlying participants’ priorities as articulated through their lived experience. I considered it important to collect data directly from people with DLB, given that much existing research focuses on care partner perspectives. The choice of a predominantly quantitative approach for the wider study reflected the recognition of the traditional prominence of quantitative data for informing policy and practice. However, the inclusion of an open-ended question enabled participants to articulate priorities in their own words. This approach was well-suited to the participant group, providing an accessible and low-burden way to capture personal, emotional, and contextual dimensions of treatment values that were not captured in the quantitative strand of the study. The data generated illustrate the added value of qualitative analysis, showing why the priorities identified in the quantitative data mattered and revealing the emotional, functional, and identity-related considerations that shaped treatment priorities.

For context, I approached the study with both academic curiosity and personal experience of dementia. My father had Alzheimer’s disease, and I witnessed the emotional and practical challenges it imposed. This sensitized me to the complexities of dementia and the multifaceted impact of symptoms. Through my PhD, I have also engaged with people with DLB and their care partners, learning from their experiences. I acknowledge that these personal and professional encounters could both illuminate and obscure aspects of participants’ accounts. To address this, I initially focused on semantic coding to stay close to participants’ words and revisited codes throughout the analysis. I also discussed the analysis with a co-author to identify potential instances where assumptions or personal resonance may have influenced the process.

**
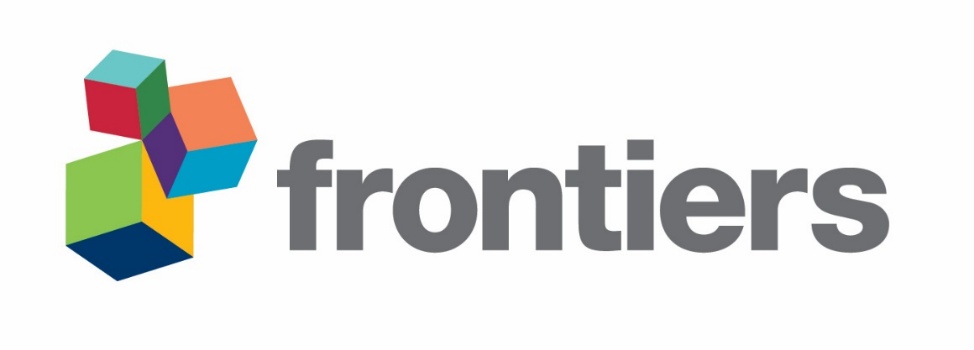
**
